# The association of food ingredients in breakfast cereal products and fumonisins production: risks identification and predictions

**DOI:** 10.1007/s12550-023-00483-5

**Published:** 2023-05-11

**Authors:** Jan Purchase, Rosa Donato, Cristiana Sacco, Lilia Pettini, Anubha Devi Rookmin, Simone Melani, Alice Artese, Diane Purchase, Massimiliano Marvasi

**Affiliations:** 1 Lux Magi Ltd., London, UK; 2 Department of Health Sciences, University of Florence, Florence, Italy; 3 Department of Natural Sciences, Middlesex University London, London, UK; 4 Department of Biology, University of Florence, Via Madonna del Piano 6, 50019 Sesto Fiorentino, Florence, Italy

**Keywords:** Mycotoxins, Fumonisins, *Fusarium*, Flour, Cereals, Decision model

## Abstract

Breakfast processed products are remarkably at risk of fungal contamination. This research surveyed the fumonisins concentration in different breakfast products and carried out in vitro experiments measuring fumonisins content in different substrates inoculated with *Fusarium verticillioides*. The pipeline started with the identification of combinations of ingredients for 58 breakfast products. Twenty-three core ingredients, seven nutritional components and production types were analyzed using a Pearson correlation, *k*-means clustering, and principal component analysis to show that no single factor is responsible for high fumonisins detection in processed cereals products. Consequently, decision tree regression was used as a means of determining and visualizing complex logical interactions between the same factors. We clustered the association of ingredients in low, medium, and high risk of fumonisin detection. The analysis showed that high fumonisins concentration is associated with those products that have high maize concentrations coupled especially with high sodium or rice. In an in vitro experiment, different media were prepared by mixing the ingredients in the proportion found in the first survey and by measuring fumonisins production by *Fusarium verticillioides*. Results showed that (1) fumonisins production by *F. verticillioides* is boosted by the synergistic effect of maize and highly ready carbohydrate content such as white flour; (2) a combination of maize > 26% (w/w), rice > 2.5% (w/w), and NaCl > 2.2% (w/w) led to high fumonisins production, while mono-ingredient products were more protective against fumonisins production. The observations in the in vitro experiments appeared to align with the decision tree model that an increase in ingredient complexity can lead to fumonisins production by *Fusarium*. However, more research is urgently needed to develop the area of predictive mycology based on the association of processing, ingredients, fungal development, and mycotoxins production.

**Supplementary Information:**

The online version contains supplementary material available at 10.1007/s12550-023-00483-5.

## Introduction

Cereal products are remarkably at risk of fungal contamination (Huong et al. 2016; Rouf Shah et al. 2016; Tola and Kebede 2016). It has been estimated that 25% of the cereals harvested annually are contaminated with mycotoxins (Eskola et al. 2018, 2020; Kamle et al. 2019). Mycotoxins are stable molecules that cannot be eliminated during the various steps of harvesting and processing. In fact, agricultural practices and inadequate drying, storage, packaging, and preservation methods can lead to an increase in the fungal load and consequently the development of mycotoxins (Liu et al. 2016). Mycotoxins can be found also in processed foods: the processing operations, such as milling, steeping, also cooking methods, and pasteurization, only partially eliminate most of the mycotoxins as they are heat resistant (Mahnine et al. 2012; Karlovsky et al. 2016; Alshannaq and Yu 2017; Kamle et al. 2019; Sarmast et al. 2021). The contaminated feed can also transfer mycotoxins to products such as milk, meat, and eggs (Alshannaq and Yu 2017).

Fumonisins are produced by *Fusarium verticillioides* and *Fusarium proliferatum* which most frequently attack maize crops worldwide. They can also be found in many other grains such as rice, wheat, barley, rye, and oats (Zentai et al. 2019; Pokrzywa and Surma 2022). Previous research has shown that *F. verticillioides* produces high levels of mycotoxins in maize and rice-derived foods at an optimal temperature between 25 and 35 °C (Huong et al. 2016; Sacco et al. 2020).

More than 15 homologs of fumonisin have been described, of these, the most commonly detected are FB_1_, FB_2_, and FB_3_ and from a quantitative point of view, FB_1_ accounts for 70–80% of the total fumonisins produced (Gil-Serna et al. 2014; IARC 2018).

According to the European Commission Regulation 1881/2006, the maximum levels for the sum of FB_1_ and FB_2_ is 1000 μg/kg in maize intended for direct human consumption (Zentai et al. 2019; Palumbo et al. 2020; Wan et al. 2020). According to the World Health Organization (WHO) and the European Food Safety Authority (EFSA), exposure to fumonisins contributes to adverse health effects, and both authorities have publicized the danger of exposure to fumonisins globally (WHO 2000; EFSA 2010; Eskola et al. 2018). According to the WHO, the daily intake for all maize-product consumers is estimated to be 0.045–0.120 µg/kg bw/day (bw, body weight). The high intake (95 percentile) ranged between 0.182 and 0.396 µg/kg bw/day, well below the 1 µg/kg bw/day tolerable daily intake (TDI) (WHO 2000). In addition, it is noteworthy that globally, there is no single strategy to avoid the risk of mycotoxin contamination (EFSA 2010; Eskola et al. 2018; IARC 2018).

One of the main safety issues with flours and some processed products is that they have a long shelf life and are routinely stored for a long time, and this can happen also for processed breakfast products. Consumers are frequently unaware of the spoilation of microfungi and mycotoxin production. As a result, efficient storage could be an effective measure against fungal growth and the production of mycotoxins. In industrialized countries, accidental fungal contamination during storage in large and small distribution and at home, although rare, cannot be excluded. There is, in fact, literature relating to the presence of mycotoxins such as fumonisins in processed and ready-to-eat products, such as breakfast cereals in European countries (Doko and Visconti 1994; Motta and Scott 2007; Cano-Sancho et al. 2012). The results of a Portuguese study showed that 96% of the analyzed breakfast cereal samples were contaminated with different mycotoxins (Martins et al. 2018). Another Portuguese study analyzed several variables including the storage conditions, the extent of the technological processing, as well as the ingredients in the finished product able to support the development of the fungal flora and the production of ochratoxin A (Duarte et al. 2010). Fumonisins in corn-based ready-to-eat foods was observed at the optimal climatic conditions for fungal development (Torović 2018). Fumonisins have been detected in processed food, for example in Morocco, where bread showed up to 133.77 μg kg^−1^ of fumonisins, or biscuits up to188.71 μg kg^−1^ (Mahnine et al. 2012). These cases may result from a combination of improperly delayed harvest and improper (home) storage (Yli-Mattila and Sundheim 2022). To our knowledge, there is no literature about the increase in mycotoxin levels during the storage of processed food.

The approach proposed here will cover all those cases where the processing operations are not enough to destroy the mycotoxin/ascospores. Firstly, a decision tree developed in this study proposes a model to predict the risk of fumonisins contamination especially focused on a mixture of ingredients. In the second part of the paper, in vitro assays show that different combinations of various ingredients are related to fungal contamination and fumonisins production. Even if the subsequent contamination of fungi producing mycotoxins may be rare, it should not be excluded as inappropriate storage (at home or in some developing countries) can occur. This proof-of-concept research shows that in the tested conditions, no single ingredient is responsible for the high fumonisins concentration, rather, the certain combination of ingredients would lead to higher fumonisins production.

## Materials and methods

### Breakfast products samplings

Fifty-eight different products for breakfast were purchased in local grocery stores (Florence municipality, Italy) in 2019 and used within the expiry date. The criteria used to purchase the products for breakfast were the following: (i) packaging size from 200 to 500 g with plastic and non-plastic packaging materials; (ii) products purchased from retail shops; (iii) any product based on wheat, maize, dry fruits, rice, and oat were chosen (Supplementary Material S1 for all ingredients). The products were classified according to (1) the type of crop, (2) if organic or not organic, and (3) packaging size expressed in weight. Products were stored in closed plastic bags, in a dark and dry cabinet at room temperature. All ingredients and nutritional facts were obtained from the labels (Supplementary Materials S1).

### Measurement of fumonisins

Breakfast products were grinded with a homogenizer Krups (Germany). The measurements were performed by adopting the enzyme-linked immunosorbent assay (ELISA method) using two kits: Defume03 (Demeditec Diagnostics GmbH, Germany) and Celer FUMO HU40032 (Eurofins Tecna, Italy) according with the user’s manual. The reagents used were 70% methanol (p.a. 99,8%- Sigma-Aldrich-MO-USA) and analytical grade water (conductivity = 1 µS/cm). An automated microplate washer (Wellwash-Thermofisher Scientific, Vantaa, Finland) was used for the washing phases. The measurements were performed with a microplate reader (Infinite F50- Tecan- Salzburg, Austria) at wavelength = 450 nm. Analytical data were analyzed with the standard curve (B/Bo% on *Y* axis, log µg/g on *X* axis) as recommended by the manufacturer’s manual. The limits of quantification of the Defume03 and Celer Fumo ELISA kits were 0.05 and 1 µg/g respectively, and the range of the standards was 0.05–5 and 1–60 µg/g. Cross reactivities for fumonisins B_1_, B_2_, and B_3_ were respectively 100%, 124 ± 11%, and 100 ± 10% for the first kit and 62% for fumonisins B_2_ for the second kit. Recovery was between 97 and 107%.

### Statistical analysis and decision model development

Principal component analysis (PCA) and *k*-means clustering were used during this research to determine the relationship between cereal ingredients, composition and packaging (the *factors*), and fumonisins concentration (the *label*). Early results indicated the possibility that concentration was associated with interacting, non-linear combinations of factor values. To investigate this possibility and determine those factor values most associated with high concentrations, a decision tree regressor was used. Regression trees are a non-parametric learning machine (James et al. 2012) that attempts to define a number of orthogonal regions in the value space of the factors of a dataset that are associated with tightly banded values of the quantitative label (concentration in this case). These regions are defined by rules (inequalities), each concerning one factor of the input data. These rules are assembled into a decision tree which can be used both to define the interaction of factors associated with different values of the label and predict the median concentration for previously unseen cases. In short, the decision tree can explain the factors high-concentration cases have in common and predict new cases.

The tree is formed by recursive binary splitting (RBS). Starting with an arrangement of all data as points in an *n*-dimensional space (*n* being the number of factors) according to the values of its factors and each having a label, this state space was successfully divided into two sub-regions. For each division, any residual regression error arising from the divided regions is less than that associated with the original, whole region. The process is binary because each division divides the existing region into two sub-regions. It is recursive because, until the stopping criterion is met, each sub-region created by the process becomes a target for further division by the same process. Each stage of binary splitting determines *one* input factor and a boundary value (of that factor) that splits the current region into two causing the greatest possible reduction in the residual sum of squares RSS.

These two sub-regions of a dataset *X*, *R*_1_, and *R*_2_ are defined, for a specified factor *j* and a pivot value *s*:

$${R}_{1}\left(j,s\right)=\left\{X|{X}_{j}< s\right\};\;{R}_{2}\left(j,s\right)=\left\{X|{X}_{j}\ge s\right\}$$

where the values *j*, *s* minimize (at each stage):

$$\sum\limits_{i:{x}_{i}\in {R}_{1}(j,s)}{\left({\widehat{y}}_{i}-{\widehat{y}}_{{R}_{1}}\right)}^{2}+\sum\limits_{i:{x}_{i}\in {R}_{2}(j,s)}{\left({\widehat{y}}_{i}-{\widehat{y}}_{{R}_{2}}\right)}^{2}$$

Here ${\widehat{y}}_{i}$ is the predicted value of the label of data point ${x}_{i}$ and ${\widehat{y}}_{{R}_{\mathrm{n}}}$ is the predicted value of the label in region *n*.

In this experiment, the Scikit-Learn (Pedregosa et al. 2011) “DecisionTreeRegressor” was used. The build parameters of the trees were as follows: depth capped to four nodes, the minimum samples per leaf were *at least* four and no pruning, and regularization or data scaling was used. Unpruned decision trees tend to overfit and yield high variance. To mitigate these risks, 75% of the data to produce (train) the tree to a bin accuracy of 96.6% was used and then classified the held-out set with a bin accuracy of 96.4%. Furthermore, the variation of factor importance for tree depths between three and 20 yielding only one pair of variant factors was tested: *Rice* and *total-fat*. All analysis was conducted using Python 3.9 and the following packages: Jupyter 1.0.0, Scikit-learn 1.0, Numpy 1.12.2, and Pandas 1.3.4. Visualizations were achieved with Matplotlib 3.4.3, Seaborn 0.11.2 and Plotly 5.4.0. A copy of Jupyter Notebook is available on request.

ANOVA was performed with Prism Software version 9.

### The Fusarium strain used in this study

The strain *F. verticillioides* GP1 was previously isolated from amaranth flour and characterized via ITS sequencing in Sacco et al. (2020). To further confirm the taxonomical classification of the GP1 isolate, in this study, the following genes were sequenced: the translation elongation factor 1-alpha, EF1 (Fw: 5′-GCYCCYGGHCAYCGTGAYTTYAT-3′; Rev 5′-ACHGTRCCRATACCACCRATCTT-3′), and ITS-LR (Fw: 5′-ACCCGCTGAACTTAAGC-3′; Rev 5′-CCGTGTTTCAAGACGGG-3′). The two genes were amplified via PCR conditions consisted of an initial denaturing step of 3 min at 95 °C, followed by 30 cycles of 95 °C for 45 s, 55 °C for 40 s, then 72 °C for 1 min for both primers. The reaction was completed with a final extension at 72 °C for 7 min. PCR was carried out with 2.5 unit of PCR Biosystem polymerase (PCR Biosystem, London, UK) on a MyCycler thermocycler (Bio-Rad, Hercules, USA). A small amount of all PCR products was analyzed by electrophoresis on 0.8% agarose gel in TBE buffer. All PCR products were sent for sequencing and identified by using the amplification products via BLAST (NCBI). Sequences are available at the NCBI Database Number (LR) ON677854 and (EF1) ON685881.

### In vitro assays for the production of fumonisins

Agar plates were prepared using different ingredients as shown in Table 1. Ingredients were purchased at the local grocery store (Florence, Italy). Sabouraud Dextrose Agar (SDA) (Oxoid, Hampshire, UK) was used as a control. All media were autoclaved and poured into Petri plates 9 cm, except for media with 30% maize flour that were directly prepared in 9 cm glass Petri plates (Table 1). An ascospore suspension of *F. verticillioides* GP1 strain was serially diluted to obtain a maximum of 5 well-separated colonies per plate when plated onto different agar media. The seeded plates were incubated at 30 °C for 4 or 6 days depending on the experiment (incubation time is reported in the figure’s caption). Once colonies were developed, each colony was then cut with a sterile cork borer of 1.2 cm diameter, it was placed in a 13-mL tube, and immediately frozen for fumonisins measurement as described above by using the ELISA test. As a control, each single flour (Table 1) used in the in vitro tests was preventively tested for basal fumonisins content, showing negligible detection: white flour 0.07 µg/g, rice flour 0.01 µg/g, and maize flour 0.27 µg/g.

**Table 1:** Media composition used in this study^a,b^

**Medium**	**Ingredient 1**^c^** (%w/v)**	**Ingredient 2**^b^** (%w/v)**	**Ingredient 3**^b^** (%w/v)**
WF4	White flour 4%	/	/
C4	Maize flour 4%	/	/
OF4	Organic flour 4%	/	/
WFM13	White flour 1%	Maize flour 3%	/
WFM22	White flour 2%	Maize flour 2%	/
WFM31	White flour 3%	Maize flour 1%	/
CRN500	Maize flour 5%	Rice flour 0%	NaCl 0%
CRN553	Maize flour 5%	Rice flour 5%	NaCl 3%
CRN3000	Maize flour 30%	Rice flour 0%	NaCl 0%
CRN3053	Maize flour 30%	Rice flour 5%	NaCl 3%
R4	Rice flour 4%	/	/
RM13	Rice flour 1%	Maize flour 3%	/
RM22	Rice flour 2%	Maize flour 2%	/
RM31	Rice flour 3%	Maize flour 1%	/

^a^Granulometries for flours were measured: whole flour 22 ± 1 µm, white flour 20 ± 1 µm, maize flour 67 ± 19 µm, rice flour 38 ± 7 µm

^b^Agar 1.5% (v/w) was added in all media except when corn flour was at 30% since the medium was already very solid

^c^All flours/corn ingredients were purchased at the local grocery store, except NaCl (Sigma-Aldrich)

### Determination of *F. verticillioides* biomass and titer of ascospores

Agar plates were prepared by using different ingredients as shown in Table 1. *F. verticillioides* GP1 biomass was determined by measuring the diameter of well-isolated fungal colonies obtained from serial dilutions (one or maximum two well-separated colonies per plate) and after 4 days of incubation. The diameter of the colonies was measured with a ruler.

With reference to the titer of the ascospores, colonies were obtained as done for the biomass production. The colonies were cut with a sterile cork borer of 1.2 cm diameter; the resulted agar was resuspended in a falcon tube with 20 mL of sterile water. Ascospores were counted using a Bürker pattern cell counting chamber (Balubrand, Merch, Darmstadt, Germany).

## Results

**Fig. 1:**
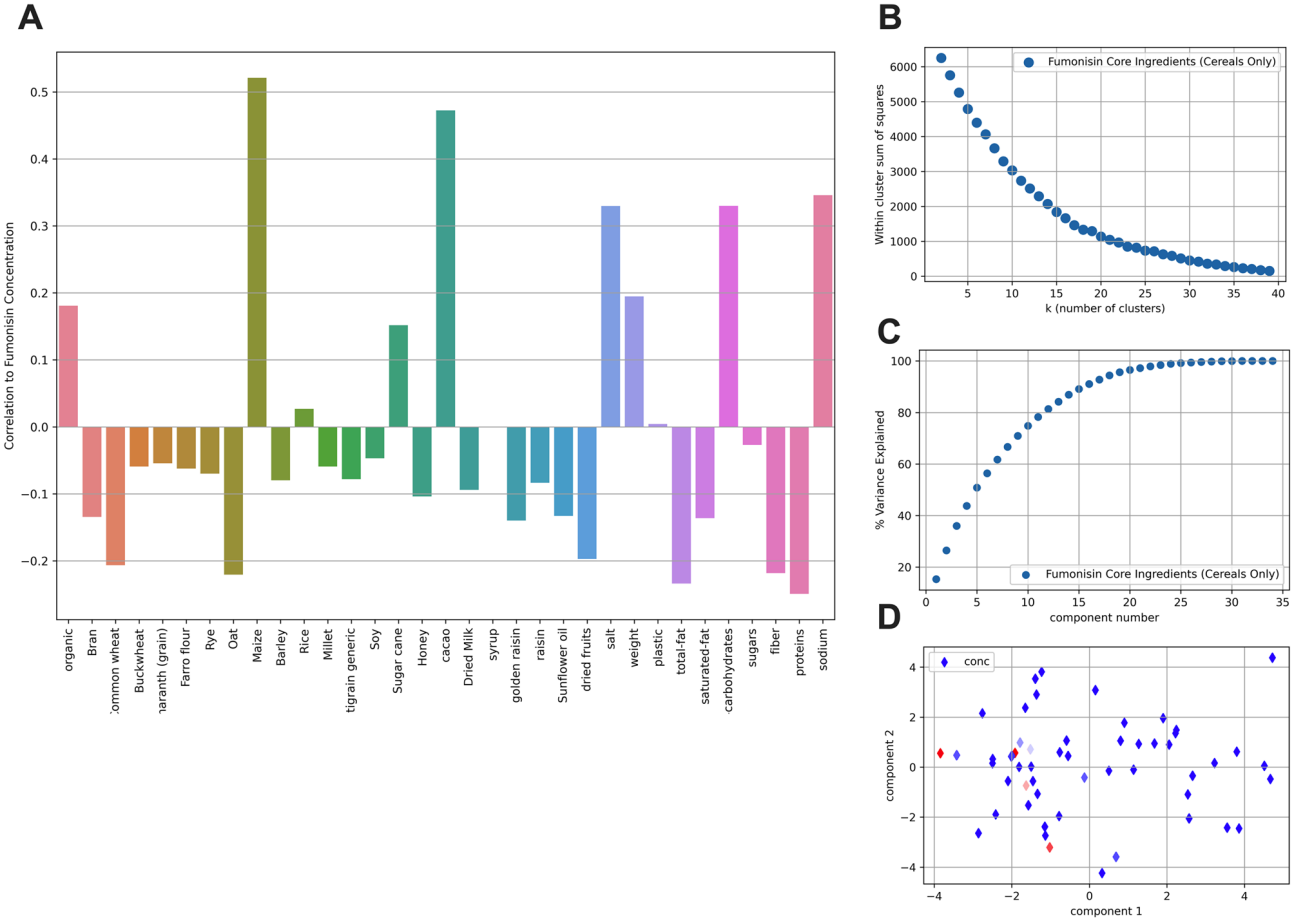
Association of fumonisins detection in ingredients of 58 breakfast products. By using product labels, the core ingredients, nutritional components, and packaging/growing types were extrapolated. **A** Pearson correlation plot between variables (core ingredients, nutritional components, and packaging/growing types) and fumonisins detection in respective products. **B** *k*-means analysis. **C** Principal component analysis (PCA). **D** Two-dimensional and color coded PCA analysis. The continuous dark blue-green-yellow color scale depicts rising levels of fumonisins concentration; dark blue is trace and yellow 160 µg/g

The ingredients described in the nutritional fact labels of 58 breakfast products were extrapolated to obtain a list of ingredients and other variables (Supplementary Material S1). These factors included the concentration of 23 core ingredients (e.g., bran, maize, rye, oats, rice, Supplementary Materials S1 and S2), seven nutritional components (total fat, saturated fat, carbohydrates, proteins, sodium, sugars, and fiber), whether the cereals were organically or non-organically cultivated, package weight and whether the packaging contained plastic or not. A Pearson correlation plot (Fig. 1A) showed no simple relationship between any one factor and fumonisins concentration (the highest correlation being with maize at 0.52). In other words, no single factor is responsible for the high fumonisins concentration detected in some cereal-based products.

**Fig. 2:**
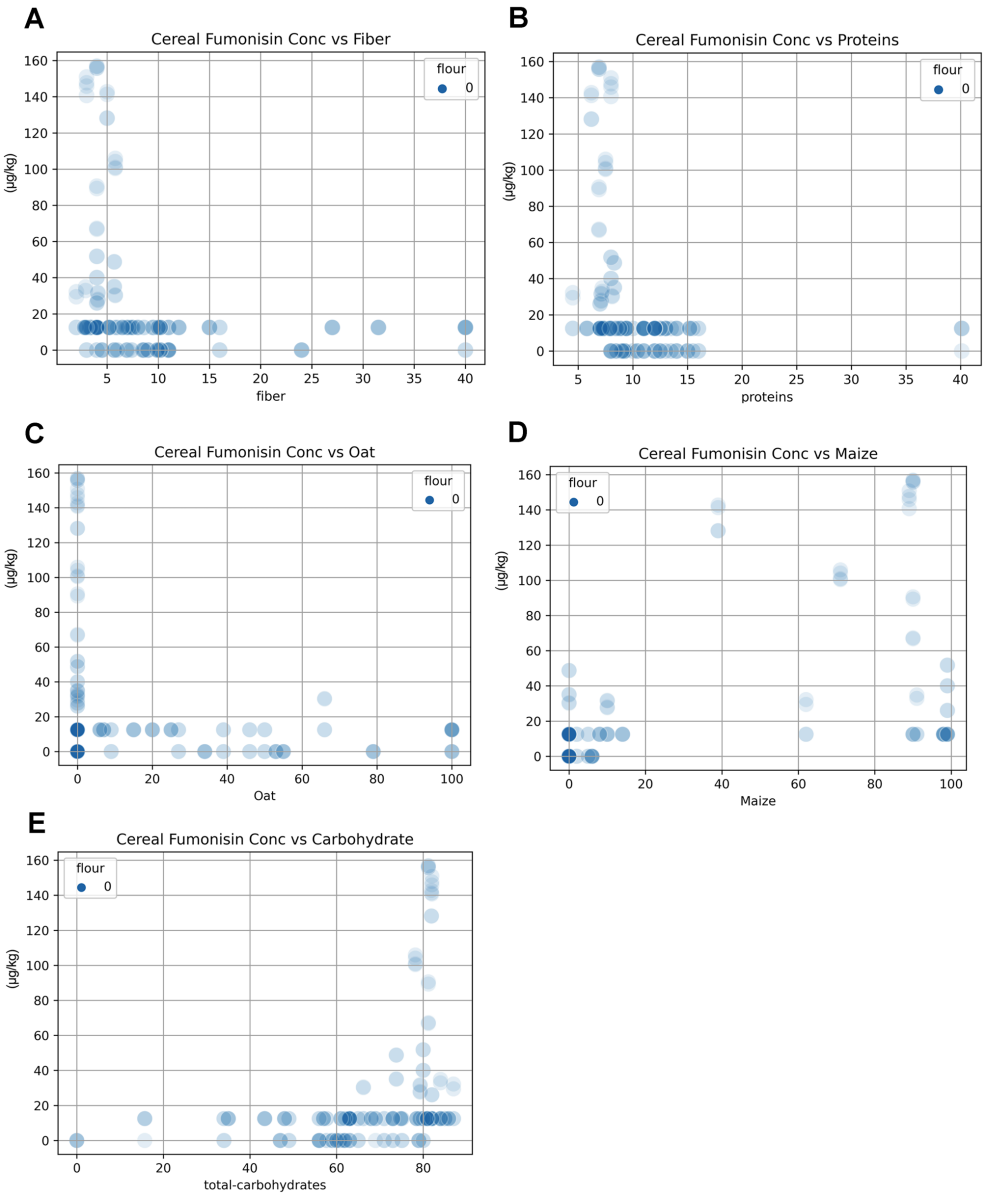
Scatter plots for the association of different ingredients with fumonisin contents. Variables are indicated on each panel. Blue circles represent different products. Color indicates data density. A light shade of blue shadow represents single data points, and deeper blue results from the overplotting of many points

A *k*-means analysis revealed no well-defined clustering of the cereals (Fig. 1B), although principal component analysis (PCA, Fig. 1C) revealed that just 11 of the factors could explain 80% of the variance in the concentration. Reduced to two dimensions and color coded by concentration, the PCA plot (Fig. 1D) shows three clusters of high fumonisins concentration aligned to elevated sodium chloride levels. However, the lack of structure or localization indicates a more complex interaction between the factors and fumonisins concentration. This is supported to some extent by the pattern of enhancement and inhibition of fumonisins concentration revealed by the scatter plots, especially maize, fibre, oats, sodium chloride, and proteins (Fig. 2). Two trends were detected: one where low content in fibers, proteins, and oat was associated with high fumonisins detection (Fig. 2A–C). A second trend, where high content in maize and total carbohydrate was associated with high fumonisins content (Fig. 2D, E).

**Fig. 3:**
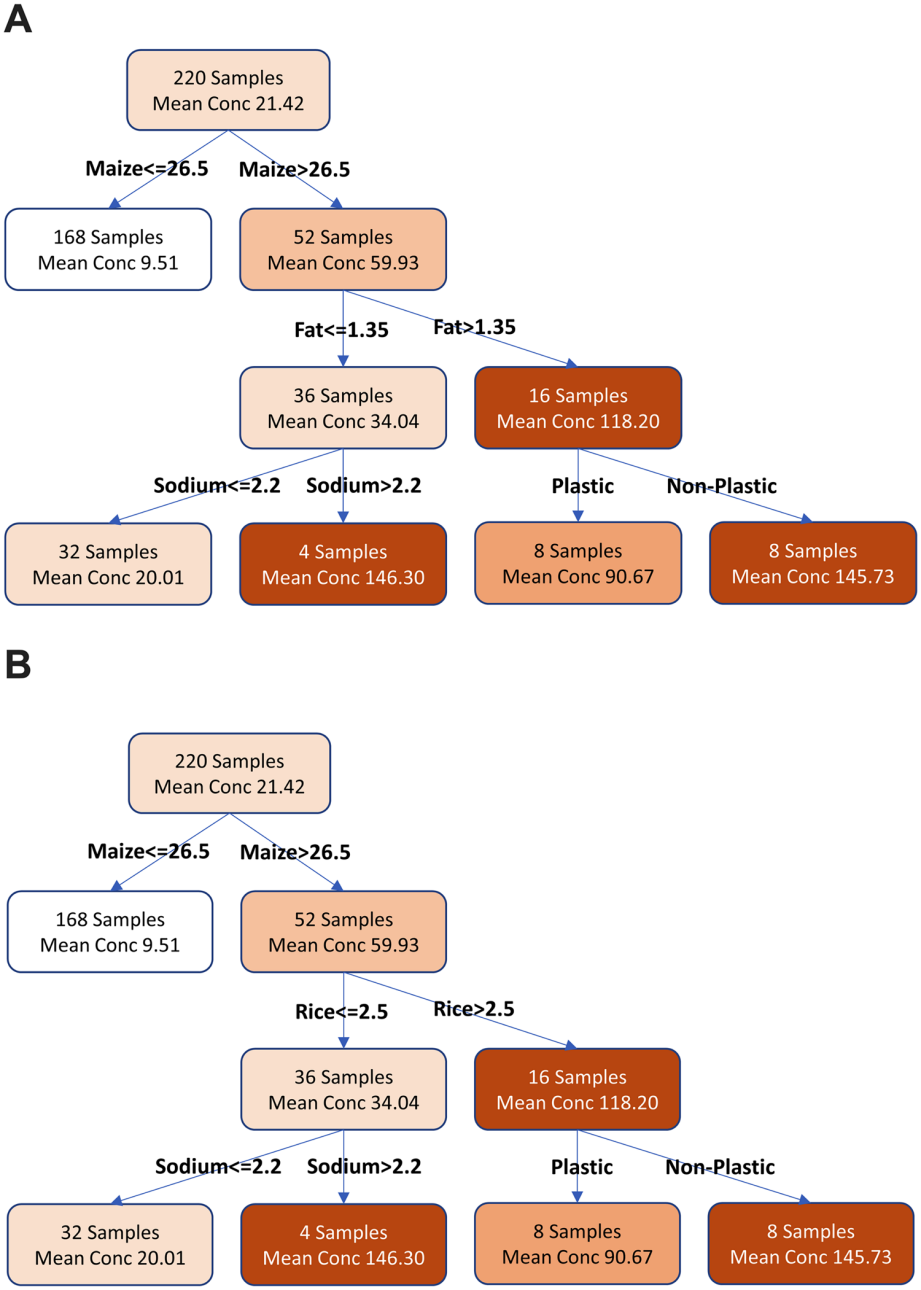
Decision tree regressions. Two analyses are proposed with similar predictive power. **A** Maize-Sodium-Rice and **B** Maize-Sodium-Fat. Heat map color shows high fumonisins content (dark orange boxes) versus low fumonisins content (white boxes). To read the decision tree start at the top and take the path dictated by the ingredient levels encountered (measured in % w/v)

Decision tree regression is an effective means of determining (and visualizing) complex logical interactions between factors that are predictive of a specific continuous quantity. Here, the application of a decision tree is proposed as a predictive model of fumonisins production. Using this approach yields a decision tree which defines a candidate rule set for determining fumonisins concentration from the factors listed above. The decision tree generated from this process (Fig. 3) suggests that fumonisins concentration is associated with cereals products that have high maize concentrations coupled especially with high levels of sodium or rice (Fig. 3A). A second tree showed maize in association with high sodium or high-fat concentrations (Fig. 3B). For both trees (right-hand side of Fig. 3A), plastic packaging counters the fumonisins concentration to some extent.

As a second part of this study, in vitro tests were performed to determine the production of fumonisins under different growth conditions. The taxonomical classification of *F. verticillioides* GB1 was confirmed by further sequencing EF1 and ITS-LR. The analysis performed via Blast (NCBI) showed 100% similarity for all the genes, belonging to *F. verticillioides.* The strain was therefore used for further in vitro tests.

**Fig. 4:**
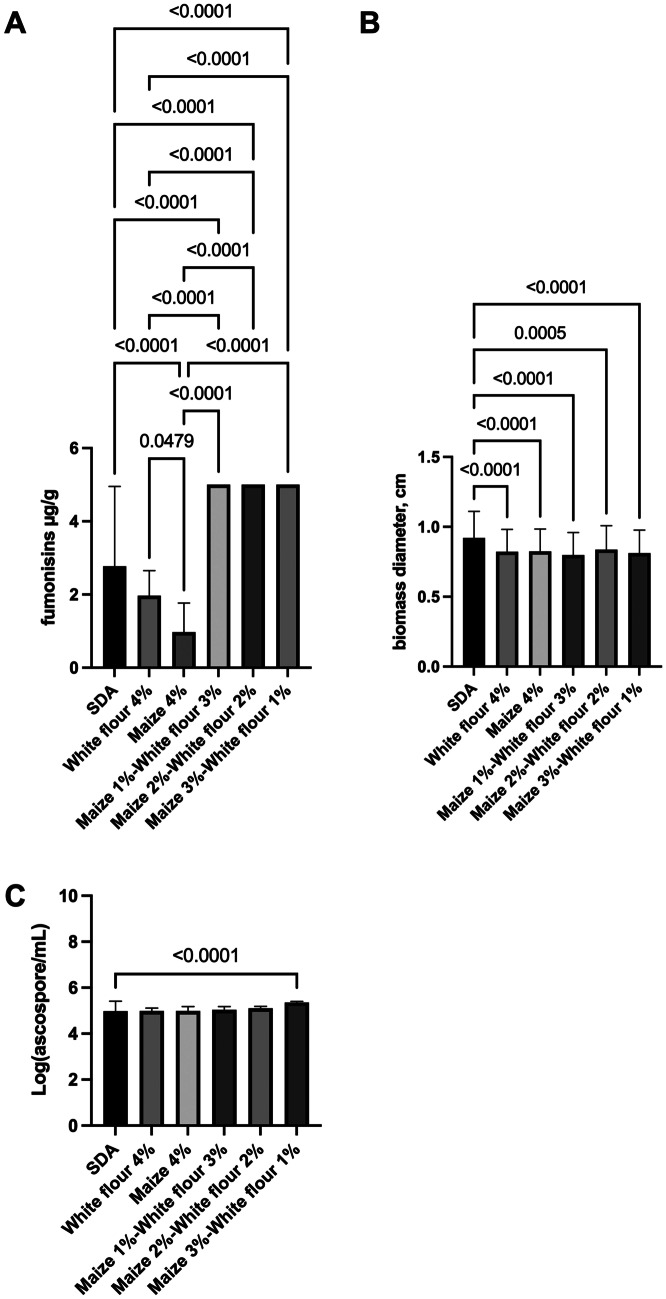
Fumonisins, biomass, and ascospores production by *F. verticillioides* on different media. *Fusarium* was incubated onto plates for 4 days. Media contain different concentrations of maize, white flour. SDA: Sabouroud agar medium. Error bars represent the standard error. Horizontal bars show the pairwise significance with a level 0.05. *p* < 0.05

The goal was to examine the extent relative proportion of maize and other carbohydrates (white flour was used as a proxy for carbohydrate, as it was called a generic ingredient in the tested breakfast products) that were associated with high fumonisins production (Fig. 4). The results showed a synergistic effect of maize and white flour measuring higher fumonisins content when compared with the single ingredients, supporting the results obtained from the distribution analysis, where no single factors are responsible for high fumonisins detection. All values for “Maize 1%-White flour 3%,” “Maize 2%-White flour 2%,” and “Maize 3%-White flour 1%” were above the upper detection limit of the kit (Fig. 4A). Fungal biomass and ascospore production for each colony on different media was also measured (Fig. 4B, C). No major differences were reported for biomass production. Also, ascospores production was constant in different media, with exception of “Maize 3%-White flour 1%,” which showed higher spore production when compared with SDA (4.9 and 5.3 log(ascospore/mL), for SDA and “Maize 3%-White flour 1%,” respectively).

**Fig. 5:**
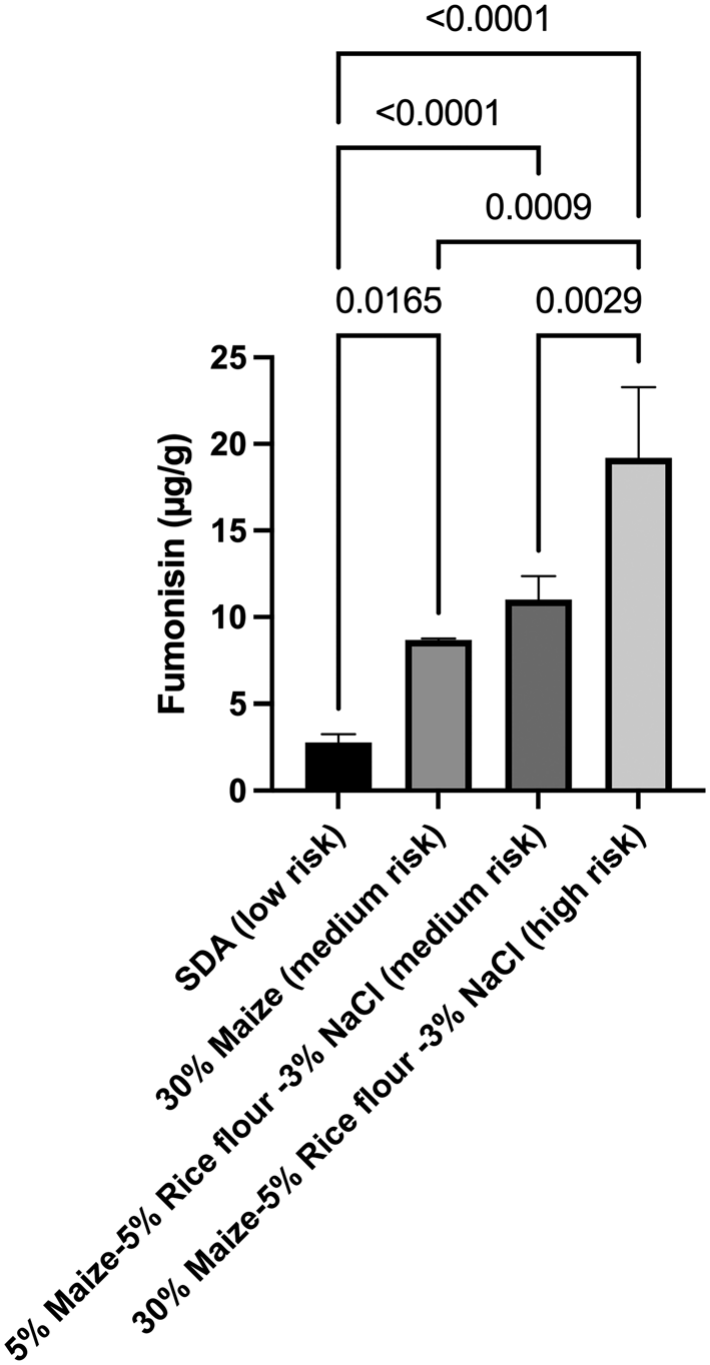
Fumonisins production by *F. verticillioides* on different media as a result of the decision tree regression. *F. verticillioides* was incubated onto plates for 6 days. Media contain different concentrations of maize, rice flour, and NaCl. SDA: Sabouroud agar medium. Error bars represent the standard error. Horizontal bars show the pairwise significance with a level 0.05. *p* < 0.05

Based on the decision tree regressions in Fig. 3A, a pool of combinations of ingredients that were at high and low risks of fumonisins content was selected (Fig. 5). The association of maize > 26% (w/w), rice > 2.5% (w/w), and NaCl > 2.2% (w/w) was considered at high risk of the presence of fumonisins (Fig. 5).

Based on the above observations, it is proposed that the model and relative levels of ingredients could be confirmed by an in vitro experiment to verify that the same combination led to a similar trend as obtained in the model prediction (Fig. 5). The results showed that fumonisins content was low in SDA 2.7 µg/g (low-risk due to the absence of maize, rice, and NaCl) when compared with medium-risk combinations 7.7 µg/g “30% maize” and 11.0 µg/g for “5% maize-5% rice flour -3% NaCl.” Interestingly, the high-risk prediction agreed with the in vitro experiments, with the highest value of 19.1 µg/g fumonisins for “30% maize-5% rice flour -3% NaCl.”

## Discussion

The total fumonisins content of fifty-eight different breakfast products purchased in different grocery stores was associated with the products’ single ingredients. The models inferred on breakfast products showed that (1) no single factor is responsible for the high fumonisins concentration and (2) the decision tree regression showed that high fumonisins concentration is associated with cereals products that have high maize concentrations coupled especially with high levels of sodium, rice, or high-fat concentrations.

The in vitro experiments also showed the effect of fumonisins production when mixing maize with other ingredients in different combinations. It is well known from the literature that mycotoxins production is differentially regulated by different carbon sources: aflatoxin production, for example, is significantly influenced by the existence of husks in the wheat forms used as carbon substrates; *Fusarium* species have been proved to produce different amounts of fumonisins if cultivated on sucrose or mannose (Li et al. 2017), starch, amylose, maltose, or glucose (Achimón et al. 2019). The literature reports that the fumonisins production is in general lower when a simple source is available (for example glucose versus kernels) (Li et al. 2017; Wu et al. 2019; Achimón et al. 2019). This is not surprising, as mycotoxins are secondary metabolites produced by *Fusarium* to increase its fitness in the surrounding environment. Fumonisins production is therefore connected to a stage in maize development (Bluhm and Woloshuk 2005), simple sugars may indicate that a specific stage is not still ready, or that ready-to-eat simple carbon sources (sugar monomers) are abundant and there is no need to activate an expensive secondary metabolite pathway. The insoluble amylopectin, for example, induces fumonisin B_1_ production in *F. verticillioides* significantly more when compared to amylose, dextrose, glucose, or maltose-containing media (Flaherty et al. 2003; Bluhm and Woloshuk 2005). Perhaps food pH also has an important role in fumonisin B_1_ production, as biosynthesis seems repressed under alkaline conditions (Flaherty et al. 2003). In this context, our results agree with the literature, where the association of ingredients may support higher levels of fumonisins (Li et al. 2017; Wu et al. 2019; Achimón et al. 2019).

In literature, other synergistic effects related to mycotoxins production have been found, but they are not related to ingredients, e.g., nisin and propionic acid were detected in aflatoxin production (Pastern et al. 1999) and plant extracts inhibit aflatoxin production in *Aspergillus flavus* (Sidhu et al. 2009). Nevertheless, such compounds can be used as food additives in marketed products to control fumonisins development and fungal growth.

In terms of the association of biomass and fumonisins production, of different *Fusarium* strains and the quantity of fumonisins produced (Melcion et al. 1997b). Literature seems to support the finding of this study, where fumonisins production is independent of biomass. Since no major differences were observed in biomass production, this suggests fumonisins are probably ascribed due to higher metabolic activity by equal biomasses. In the literature, in vitro production of fumonisins according to the fungal biomass has been found to mainly change after 15 days of incubation at 30 °C on potato-dextrose-agar (PDA) (Melcion et al. 1997a). These findings may suggest that products made with single ingredients such as maize only could delay fumonisins synthesis. Further tests can be done with different concentrations and ingredients being typical of processed food, e.g., salt, different starch polymers, and preservatives may lead to different production of fumonisins.

In this context, further research should be carried out: (1) to generate literature about the increase in mycotoxin levels during storage of processed food, as there is a complete lack of information, and (2) to develop a model that can predict to what extent ingredients (and their mixtures) support fungal growth and mycotoxin production. In our specific case, the results from the in vitro assay cannot be used to predict mycotoxin contamination during storage; however, the interesting aspect is that the models and the in vitro observation agree: the increase of ingredient complexity seems to lead to higher fumonisins content. Once developed these models, they can be applied to risk assessment. After package opening, during domestic storage, cereals absorb a considerable amount of humidity which worsens products’ organoleptic and nutritional quality and the percentage content of water can be much higher, especially in mixed products such as muesli. The increase of water can therefore increase the risk of fungal growth.

From a risk assessment perspective and modelling, it is well known that the crops may reach the processing factory already heavily contaminated by *Fusarium* spp. (Folcher et al. 2009) and models are already proposed in this field to improve best practices and prevent crop contamination on the field (Maiorano et al. 2009). Different simulation models have been proposed: (1) to predict pre-harvest aflatoxin risk in maize according to cardinal temperatures of *Aspergillus flavus* and drought index (Chauhan et al. 2015), (2) to model the production of aflatoxins during active growth and stationary phase of *A. flavus* on maize (Garcia et al. 2013), and (3) to model aflatoxins production by *A. flavus* as a function of water activity and temperature on polished and brown rice (Mousa et al. 2013). Other interesting fields for modelling are the effect of climate change on aflatoxins production and storage parameters (Medina et al. 2014; Akinola et al. 2021). Further models have been developed to predict and prevent mold spoilage of food products (Dagnas and Membré 2013).

In conclusion, this paper suggests that an increase in ingredient complexity can lead to fumonisins production by *Fusarium*. This prompts a further question, “can the combination of food ingredients be further used to control fumonisins production?” Future work is recommended in the area of predictive mycology based on the association of processing, ingredients, fungal development, and mycotoxins production at a higher resolution.

## Supplementary Information

Below is the link to the electronic supplementary material.

Supplementary file1 (XLSX 27 KB)

Supplementary file2 (XLSX 12 KB)
